# Plant‐driven changes in soil microbial communities influence seed germination through negative feedbacks

**DOI:** 10.1002/ece3.5476

**Published:** 2019-07-25

**Authors:** Elizabeth C. Miller, Gabriel G. Perron, Cathy D. Collins

**Affiliations:** ^1^ Biology Program Bard College Annandale‐on‐Hudson NY USA

**Keywords:** fungal pathogen, host specialization, ITS sequencing, metagenomics, plant–pathogen interactions, plant–soil feedback, seed germination, soil microbial community

## Abstract

1. Plant–soil feedbacks (PSFs) drive plant community diversity via interactions between plants and soil microbes. However, we know little about how frequently PSFs affect plants at the seed stage, and the compositional shifts in fungi that accompany PSFs on germination.
2. We conducted a pairwise PSF experiment to test whether seed germination was differentially impacted by conspecific versus heterospecific soils for seven grassland species. We used metagenomics to characterize shifts in fungal community composition in soils conditioned by each plant species. To investigate whether changes in the abundance of certain fungal taxa were associated with multiple PSFs, we assigned taxonomy to soil fungi and identified putative pathogens that were significantly more abundant in soils conditioned by plant species that experienced negative or positive PSFs.
3. We observed negative, positive, and neutral PSFs on seed germination. Although conspecific and heterospecific soils for pairs with significant PSFs contained host‐specialized soil fungal communities, soils with specialized microbial communities did not always lead to PSFs. The identity of host‐specialized pathogens, that is, taxa uniquely present or significantly more abundant in soils conditioned by plant species experiencing negative PSFs, overlapped among plant species, while putative pathogens within a single host plant species differed depending on the identity of the heterospecific plant partner. Finally, the magnitude of feedback on germination was not related to the degree of fungal community differentiation between species pairs involved in negative PSFs.
4. *Synthesis*. Our findings reveal the potential importance of PSFs at the seed stage. Although plant species developed specialized fungal communities in rhizosphere soil, pathogens were not strictly host‐specific and varied not just between plant species, but according to the identity of plant partner. These results illustrate the complexity of microbe‐mediated interactions between plants at different life stages that next‐generation sequencing can begin to unravel.

## 1 INTRODUCTION

Plant species influence soil microbial communities, which in turn alter subsequent plant growth—a phenomenon known as plant–soil feedback (PSF; Bever, 1994). Negative PSFs occur when plants accrue less biomass or suffer lower fitness in soils inhabited by the same species compared to soils inhabited by a different species (Bever, 1994). Negative PSFs are driven in large part by host‐specific pathogens (Crawford et al., 2019) and have been implicated as a key mechanism for generating and maintaining plant diversity in tropical forests (Mangan et al., 2010), temperate forests (Packer & Clay, 2000), and temperate grasslands (Petermann, Fergus, Turnbull, & Schmid, 2008). In contrast, positive PSFs can decrease plant diversity when individual plants perform better near plants of the same species, such as when fungal mutualists provide a competitive advantage to later‐successional host plant species (Cortois, Schröder‐Georgi, Weigelt, Putten, & Deyn, 2016; Kardol, Bezemer, & van der Putten, 2006). Despite strong support for the importance of PSFs for structuring plant communities, two key gaps in our understanding remain: (a) the degree to which seeds (as opposed to seedlings or adult biomass) are impacted by negative PSFs and (b) the magnitude of compositional shifts in soil microbial communities, both over time and among host plant species, needed to sustain PSFs.

Most experimental evidence for PSFs comes from studies on plant biomass (Kulmatiski, Beard, Stevens, & Cobbold, 2008). However, seeds represent a key component of fitness for flowering plants. Mortality during the seed‐to‐seedling transition can lead to demographic bottlenecks (Harper, 1977), limiting plant establishment (e.g., James, Drenovsky, Monaco, & Rinella, 2011) and local plant diversity (Grubb, 1977). Seeds of many grassland, tropical forest pioneer, and woodland herbaceous species can survive in the soil for years or even decades, facilitating long‐term population persistence via recruitment into tree gaps or local soil disturbances (Dalling & Brown, 2009; Jankowska‐Blaszczuk & Grubb, 2006; Kalamees & Zobel, 2002). Soil‐borne pathogens are a key agent of seed mortality for buried seeds (Gallery, Moore, & Dalling, 2010; Kirkpatrick & Bazzaz, 1979; Sarmiento et al., 2017), suggesting that seeds may be vulnerable to pathogen‐mediated negative feedbacks.

Evidence for the effects of PSFs emerging from the few seed‐focused studies remains mixed. For instance, host‐specialized pathogens selectively decrease germination in tropical tree species (Gallery, Dalling, & Arnold, 2007; Sarmiento et al., 2017), consistent with the notion that negative PSFs affect plant success before seedling establishment. In contrast, a recent study of three grassland herbaceous species detected only neutral PSFs at the seed stage (Dudenhöffer, Ebeling, Klein, & Wagg, 2018). If negative PSFs reduce seed survival near conspecific plants, the impact of PSFs on plant population persistence, community composition, and diversity maintenance is likely underestimated.

PSFs occur because plant species alter the composition of fungal and bacterial communities in soil by releasing exudates and other compounds from roots (Bever, Platt, & Morton, 2012). Recent metagenomic analyses of soil microbial communities have shed new light on the influence of plant traits on microbial communities and associated PSFs (Kulmatiski et al., 2017) and the role of PSFs in succession following the loss of dominant tree species (Pfennigwerth, Van Nuland, Bailey, & Schweitzer, 2018). However, these techniques have not yet been used to characterize shifts in microbial communities as different plant species “condition” soil over time, or to link compositional changes with subsequent impacts on seed germination. Because pathogen‐induced seed mortality does not necessarily reflect negative PSFs (e.g., when generalist pathogens predominate and kill seeds regardless of plant identity), establishing host specialization and identifying putative pathogens alongside differences in germination success will help demystify the “black box” of belowground biota implicated in driving PSFs.

In particular, a key question that remains largely unaddressed is the extent to which various PSF relationships are the result of large‐scale shifts in entire soil communities versus changes in a few influential microbes (Benítez, Hersh, Vilgalys, & Clark, 2013). Given that plant‐induced changes to soil microbial communities likely involve taxa other than pathogens, distinct, host‐specialized microbial communities will not necessarily lead to differential mortality of seeds buried in conspecific versus heterospecific soils. For instance, microbial communities in soils surrounding individual plants may differ primarily due to the composition of saprotrophic fungi present in each, even though they possess similar pathogens and mutualists. Moreover, pathogens infecting multiple host species can have partner‐dependent effects (Hersh, Vilgalys, & Clark, 2012); in these cases, fungal communities may not contain unique pathogens, and we may still observe directional PSFs. Alternatively, the presence of a single, influential host‐specific pathogen might reduce seed germination in soils conditioned by conspecific plants, generating negative PSFs, while other functional guilds in the microbial community remain largely unchanged relative to heterospecifically conditioned soils. In this case, we might observe negative PSFs, but not detect host‐specialized microbial communities.

In this study, we tested whether PSFs occur at the seed stage of seven grassland species by exposing seeds to soils conditioned by conspecific and heterospecific plants. We then used high‐throughput ITS amplicon sequencing to quantify fungal communities in soils that developed over time for each plant species. Finally, we asked whether compositional differences were associated with the magnitude of PSFs on seed germination. We hypothesized that (a) seeds will succumb to negative PSFs such that fewer seeds germinate in soils conditioned by conspecific relative to heterospecific plants; (b) fungal community composition, and in particular the presence and identity of putative pathogens, will vary between soils depending on plant species identity as a result of plant conditioning; and (c) because directional feedbacks likely reflect the net outcome of the presence and interactions of numerous fungal taxa, the magnitude of the feedback between two species will correlate positively with the magnitude of compositional differences between fungal communities in their conditioned soils. We focused on fungi because fungicide studies frequently reveal negative effects of fungi on grassland seeds (Beckstead, Street, Meyer, & Allen, 2011; Mordecai, 2011; Schafer & Kotanen, 2003), making fungi reasonable candidates for driving negative PSFs on seeds in our system.

## 2 MATERIALS AND METHODS

### 2.1 Soil conditioning stage

To characterize plant‐induced changes in soil fungi and prepare soils for PSF experiments, we conditioned soils by growing individuals of seven plant species in separate pots. Soils were collected in August 2017 from the University of Kansas Field Station (hereafter referred to as KFS), located ca. 20 km north of Lawrence, Kansas, U.S.A., in the prairie–forest ecotone. We collected soils from the top ten centimeters of the soil profile in six different patches of secondary forest (each patch was 288 m^2^) embedded in a mowed grassland matrix within an experimentally fragmented landscape (for details regarding experimental landscape, see Holt, Robinson, & Gaines, 1995). Although woody species currently dominate the canopy in the patches, populations of old field and grassland species persist in tree gaps and patch edges, as well as in the mowed matrix surrounding the patches (Collins, Holt, & Foster, 2009). Thus, our site represents a successional mosaic typical of many anthropogenically altered landscapes (Vellend, 2003), and locally coexisting species include plants representing early and late successional stages. All patches from which we sampled soils contained the seven focal species in our study which are generally widespread in the landscape (Table S1). Soil samples were sieved through 1‐cm hardware cloth in the field, and soils collected from different locations in the landscape were homogenized in plastic tubs. The homogenized soil was stored at 4°C for three weeks prior to beginning the conditioning experiment. When the conditioning experiment began, we froze a portion of the collected soil at –80°C to preserve for later characterization of the baseline (“Day 0”) fungal community.

We used seven focal species (hereafter referred to by genus) in our study: two grasses (*Bromus inermis* Leyss, *Poa pratensis* L.), two asters (*Ageratina altissima* R.M. King & H. Rob.*, Solidago canadensis* L.), one legume (*Desmodium illinoense* A. Gray), one herbaceous species in the rose family (*Geum canadense* Jacq.), and one herbaceous species in the mint family (*Pycnanthemum tenuifolium* Schrad.). These species coexist in our sites at the Kansas Field Station and were chosen because they collectively represent diverse taxonomy and life history traits (Table S1). Seeds were purchased from Ernst Conservation Seeds Inc., Prairie Moon Nursery, and Sheffield's Seed Co. Inc., specifying ecotypes closest to Kansas when possible (Table S1).

We germinated plants in 273 conical plastic pots with a mixture of three parts field soil and one part sand which had been sterilized by autoclaving twice, 24 hr apart to eliminate surviving endospores. To maximize the number of racks we could house in available growth chamber space, we used two different pot sizes, hereafter referred to as “big” (Greenhouse Megastore, CN‐SS‐SC, 5.5 × 5.5 × 18 cm, 0.164 L) and “small” (Greenhouse Megastore, CN‐SS‐DP, 4 × 4 × 21 cm, 0.107 L) pots. We included 21 replicates planted in big and 14 in small pots for all species except *Desmodium*, of which 35 replicates were planted in big pots only (Table S1). We also included 21 small pots containing only field soil. We did not bury seeds in these pots during the feedback experiment (see below); rather, we used them as a “no‐plant” control to observe changes in fungal composition over the conditioning period without the influence of plants.

Plants conditioned soil for about three months (101 days for large pots and 93 days for small pots), a duration sufficient to generate species‐specific PSFs (Lepinay, Vondráková, Dostálek, & Münzbergová, 2018). Seedlings were housed in a growth chamber (Percival, Perry, IA, model AR‐66L2) on a 14:10 light:dark cycle (32‐watt bulbs), 25°C day and 20°C night, to mimic late spring conditions in Northeast Kansas. We saturated soil with deionized water every one to three days (as needed) to maintain soil moisture at field capacity.

Once most seedlings were at the second leaf stage (around 30 days), we thinned all pots to three individuals. We retained three plants per pot to prevent loss of samples in case of plant mortality. To minimize effects of species‐specific, plant‐induced changes to soil nutrients, as well as to minimize plant stress which may alter root exudates and consequently microbial communities (Bever, Platt, & Morton, 2012), we added a single application of 20 ml Miracle‐Gro^©^ nutrients to each pot (Day 83 for large pots, Day 68 for small pots), including the controls. The concentrate (12% N, 4% P_2_O_5_, 8% K_2_O, 0.10% Fe, 0.05% Mn, and 0.05% Zn) was diluted to 1.5 ml per 1 L sterile water, as per manufacturer's instructions. Although nutrient addition can cause short‐term changes in soil fungal communities (Schmidt et al., 2007), comparisons of fungal communities among plant species were likely unaffected because all plants received the same treatment. Moreover, plant identity has a stronger influence on community composition than soil nutrients (Burns, Anacker, Strauss, & Burke, 2015).

### 2.2 PSF experiment

To measure PSF effects on seed germination, we buried seeds of the seven species in soil conditioned for three months by conspecific plants, as well as by each heterospecific species, in a full factorial design. We included five replicates for each conspecific and heterospecific soil pairing. We used soil surrounding plant roots grown in three randomly chosen big pots and two randomly chosen small pots for each species except *Desmodium* (because all five replicates were grown in big pots), for a total of 245 experimental pots. Each pot of soil for the seed burial (exposure) phase contained inoculum from a different replicate plant from the conditioning phase, thereby ensuring statistical independence among experimental units.

We used sterilized tools to remove the soil from the plant roots; conditioned soil (without roots) was then used as inoculum during seed burial. We mixed one part conditioned soil with two parts sterile soil and ½ part sterile sand. This mixture was used to fill new pots, sterilized in a 10% bleach solution, in which we buried seeds. We also buried four replicates of each seed species in a combination of two parts sterile potting soil (never conditioned by plants) and one part sterile sand to quantify the overall effect of soil biota on germination (as opposed to biota conditioned by another plant species). All of the seed burial replicates for all species, including sterile soil, were in big pots regardless of soil origin during this exposure phase.

In each pot, we placed 25 seeds sealed in a nylon mesh bag (Thermo Fisher, 6774010, pore size 0.282 mm) that contained the seeds while still allowing access for microbes. To remove surface contaminants, each bag of seeds was sterilized in a 10% bleach solution (5.25% NaOCl) for 10 s then allowed to dry, before being buried in pots. We opted not to soak seeds for longer in an effort to minimize any direct, species‐specific effects of bleach on germination (Ditommaso & Nurse, 2004), at the possible cost of not eliminating all microbiota from the surface of the seed. Soil was dampened with deionized water every 7–10 days to prevent extreme changes in soil moisture shown to affect PSFs (Fry et al., 2018).

After 90 days of exposure to conditioned soil, we removed all seeds and placed them on dampened filter paper in petri dishes. Seeds that germinated prior to exhumation were categorized as successful germinants. Of those that germinated before exhumation, none were large enough to penetrate mesh bags or the soil surface, so we assumed that germination occurred after some exposure to soil microbial communities. The petri dishes were placed in a seed germination chamber (Percival, model GR‐36L) at 30°C (average June high temperature in Kansas), with 32‐watt lights on 24 hr. The filter paper was kept moist, and the arrangement of dishes in the growth chamber was randomized weekly. Seeds were checked every three days for the emergence of a radicle and were removed from the dish following germination. The experiment continued for each species until 14 days had passed without new germination (a total of 20–60 days, depending on species).

### 2.3 ITS2 rRNA sequencing

To characterize soil fungal communities, we sequenced ITS2 rRNA genes from 94 soil samples: eleven samples of soil under each plant species (except for *Desmodium*, for which there were only eight samples), ten samples of control soil conditioned without plants, and ten samples from soil preconditioning (i.e., Day 0). Day 0 replicates were subsampled from the same bulk soil sample. For plant‐conditioned soils, we used the same five soils also used as inoculum for the seed burial phase in the feedback experiment (above), as well as six additional samples randomly selected from the 30 unused replicates of plant‐conditioned soils. From each pot, we collected soil “cores” using a sterilized drinking straw and stored samples at –80°C. Soil samples were thawed and homogenized, and DNA was extracted from 0.25 g of soil using the MoBio PowerSoil DNA Isolation Kit (MoBio). DNA concentrations were assessed using the NanoDrop 2000 (Thermo Fisher), and DNA samples were stored at –20°C.

Fungi were characterized via targeted amplification of the ITS2 rRNA gene region. ITS2 amplification was performed using ITSF forward and ITS2 reverse primers with barcode sequences on the reverse (Walters et al., 2016). PCR amplification was performed as described in the Earth Microbiome Project ITS Illumina Amplicon Protocol (Walters et al., 2016), and libraries were sequenced on a MiSeq Illumina platform using the protocol for 250 bp paired reads (Wright Labs).

### 2.4 Processing and analysis of ITS2 sequence data

Fungal communities were analyzed using the DADA2 1.8 microbiome pipeline (Callahan et al., 2016; available at https://github.com/benjjneb/dada2) implemented in R 3.2.3 (R Core Team, 2017; http://www.r-project.org). Briefly, we identified forward and reverse primers on the sequencing reads and removed the primers using cutadapt (Martin, 2011). The reads were then filtered according to the following parameters: maximum of two expected errors (from the quality score) and minimum length of 50 bases. The filtered reads were dereplicated, combining all identical reads into unique sequences. We then used DADA2 to construct consensus quality scores, which were used in a denoising algorithm to infer error rates and separate sequencing errors from true sequence variation. The unique sequences, known as amplicon sequence variants (ASVs), are homologous to the operational taxonomic units (OTUs) used in traditional metagenomic pipelines, but are based on estimates of true sequence variation rather than grouping by sequence similarity (Callahan, McMurdie, & Holmes, 2017). After ASV assignment, chimeras were removed and taxonomy was assigned to each ASV using General Fasta Release Files from the UNITE ITS database (Kõljalg et al., 2005).

We processed soil community data using the phyloseq package (McMurdie & Holmes, 2013; v1.14.0; available at https://joey711.github.io/phyloseq/). To prevent rare taxa from skewing compositional and diversity analyses (below), we excluded taxa not occurring at least three times in at least 15% of samples.

We identified host‐specialized fungal taxa using the DESeq2 differential abundance comparison (Love, Huber, & Anders, 2014), adapted for use with microbial count data (McMurdie & Holmes, 2014). Specifically, we identified fungal ASVs that differed in presence or abundance between soils conditioned by species pairs involved in PSFs as indicated by statistically significant feedbacks (see below). Ecological guilds were assigned to DESeq‐identified ASVs using FUNGuild (Nguyen et al., 2016) based on UNITE taxonomic assignments (Kõljalg et al., 2005) and were broadly grouped by function into arbuscular mycorrhizae, ectomycorrhizae, endophytes, plant pathogens, and saprotrophs (for specific assignments and all ASVs, see Table S2). We used only guild assignments with a confidence factor of Probable or Highly Probable; all others were considered unassigned.

### 2.5 Statistical analysis

Germination data were analyzed and visualized in R (v.3.4.2; R Core Team, 2017), using the packages ggplot2 (v.2.0.0; Wickham, 2016), ggsignif (v.0.4.0; Ahlmann‐Etze, 2017), nlme (v. 3.1‐137; Pinheiro, Bates, DebRoy, & R Core Team, 2019), and vegan (v.2.3.2; Oksanen et al., 2018). To test for negative feedbacks on germination, we analyzed each focal species separately using proportion of seeds germinated as the response variable, and soil identity (determined by identity of plant species that conditioned soil) as a fixed effect in a general linear model. We confirmed that our data did not violate assumptions of model using Shapiro–Wilk test for normality and Levene's test for equal variances. Assumptions were met for all species except *Bromus*, for which we used a generalized linear model (GLM) with a binomial distribution. Within each species’ model, we used treatment contrasts to compare seed germination in soil conditioned by each species (hereafter heterospecific soil) relative to germination in conspecific soil by setting conspecific soil as the reference level. We performed these contrasts even when the omnibus test for the model was not significant because our interest was not simply in testing whether group means were equal; rather, we aimed to characterize pairwise species interactions. Nonetheless, all pairs in which we detected significant feedbacks were detected in models with omnibus tests yielding *p* < .1.

To visualize feedbacks in a way that allows for comparison among species and with other studies, we calculated PSFs as the natural log of the ratio between germination success in conspecific (C) and heterospecific (H) soils [ln(C/H)]; consequently, positive and negative feedbacks are symmetrical around zero (Brinkman, Van der Putten, Bakker, & Verhoeven, 2010). As pots were not paired in our experimental design, mean values for feedbacks were generated by averaging all possible conspecific and heterospecific pairings following Gómez‐Aparicio et al. (2017) and Kuebbing, Classen, Call, Henning, and Simberloff (2015). Five conspecific replicates crossed with five heterospecific replicates yielded 25 values for each species pair. We did not calculate feedbacks relative to sterile soil because our question was focused on pairwise feedbacks as they relate to host‐specialized microbial communities, not overall impact of soil biota on a single species' germination. Many authors use one‐sample *t* tests to distinguish feedbacks calculated on ratios from zero (Brinkman et al., 2010); while we include results from this approach in Supporting information, we rely on linear models—the more conservative of our approaches—for interpretation. Following our analyses of PSFs, we used a Kruskal–Wallis test to assess differences among focal species in the proportion of total germinants that germinated during the seed burial (exposure) phase prior to exhumation; our goal was to explore whether rapid germination (i.e., early escape from seed pathogen pressure) might help interpret species‐specific susceptibility to pathogen‐mediated PSFs we observed.

Fungal communities based on sequence data were characterized using Primer (Clarke & Gorley, 2001). We compared fungal community diversity among soils conditioned by different plant species and among pot sizes with a two‐way ANOVA using ASV richness as the response variable. To test whether fungal community composition varied according to plant identity and pot size, we performed a permutational multivariate analysis of variance (PERMANOVA; Primer; Clarke & Gorley, 2001) with 9,999 permutations and Type III sum of squares performed on a Morisita–Horn distance matrix calculated for abundance data (number of reads) transformed using log (*x* + 1). We chose Morisita–Horn because it is not affected by rare species and is robust to undersampling (Jost, Chao, & Chazdon, 2011). We visualized data using principal coordinate analyses (PCoAs) which project points in units of the chosen distance metric, thereby aligning the visual ordination of the statistical test for differences in community composition. We ran PERMANOVA models for data including Day 0 (to assess differentiation among species, and relative to baseline soils) as well as excluding Day 0 (to assess fungal community differentiation among soils at the end of the conditioning period). To account for multiple tests in our post hoc comparisons, we assessed significance for pairwise differences under a controlled false discovery rate (set at 0.05) following the Benjamini–Hochberg procedure (Benjamini & Hochberg, 1995). We report original *p*‐values, with bold‐faced font indicating values considered statistically significant after correcting for multiple tests.

For species pairs that generated statistically significant PSFs, we used Spearman's rank correlation to determine whether the magnitude of feedback increased with the magnitude of fungal community divergence. We did this for positive and negative PSFs, then again for negative PSFs only. For these analyses, we first used PERMANOVA to test differences in soil fungal communities between the eight species for which we detected feedbacks. These tests, along with PCoA visualizations, were conducted on a dataset that included soils conditioned in different pot sizes (and are indicated accordingly in ordinations) because soils from both pot sizes contributed to replicates in the feedback portion of the study. Community divergence was quantified using the distance between centroids (mean distance between replicate communities for a single species) of fungal communities in soils conditioned by two different plant species.

## 3 RESULTS

### 3.1 PSFs: effect on seed germination

**Figure 1:**
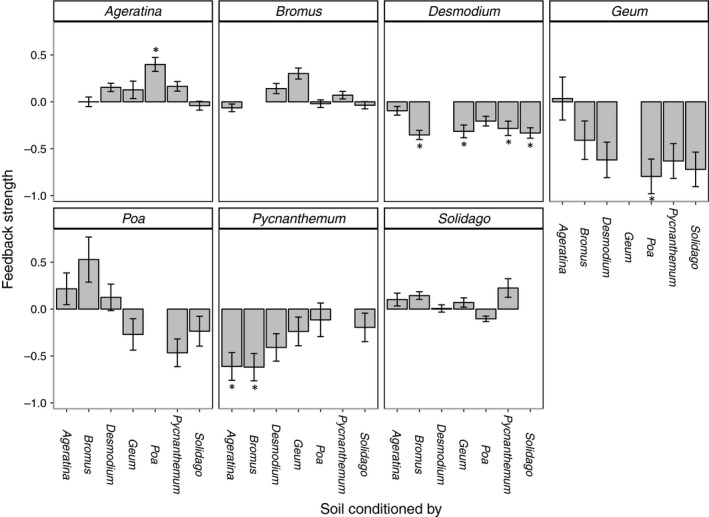
Strength of PSFs on seeds germinated in conspecific versus heterospecific soil. Each panel contains results for seeds of a different plant species; *x*‐axis labels reflect the identity of the heterospecific plant‐conditioned soil. PSFs were calculated as ln(germination in conspecific soil/germination in heterospecific soil). Positive values indicate positive PSFs (higher germination in conspecific soil compared to other species' or sterile soil), and negative feedback values indicate negative PSFs (lower germination in conspecific soil compared to other species' or sterile soil). Error bars show standard error, and * indicates statistically significant PSFs (*p* < .05) from linear models on proportion of seeds that germinated (germination data shown in Figure S2, Tables S3–S9)

To test for the effect of PSFs on seed germination in each of our seven focal species, we measured the percent of seeds that germinated after being buried for three months in conditioned soils. Timing of germination varied among species (*χ*_(6)_ = 221.6, *p* < .001; Figure S1); most seeds of both cool‐season grasses (*Bromus* and *Poa*) germinated while buried during the exposure phase (Figure S1). Overall, eight of forty‐two species pairs (19.0%) produced directional PSFs (Figure 1). Specifically, we detected seven negative PSFs in which seeds germinated less frequently in conspecific soil relative to soil from a heterospecific partner, and one case in which seeds experienced higher germination success in conspecific relative to a heterospecific soil, i.e., a positive PSF (Figure 1, Figure S2). No species exhibited negative PSFs with all heterospecific partners, although *Desmodium* germinated less frequently in conspecific soils relative to four heterospecific partners: *Geum*, *Bromus*, *Pycnanthemum*, and *Solidago* (Figure 1; Table S3). *Desmodium* also germinated more in sterile soil than in conspecific soil (Figure S2). *Pycnanthemum* experienced negative PSFs with *Ageratina* and *Bromus* (Figure 1, Table S4), and *Geum* experienced a negative feedback with *Poa* (Figure 1; Table S5). *Ageratina*, on the other hand, exhibited a positive PSF with *Poa* (Figure 1; Table S6), with higher germination success in conspecific soil relative to soil conditioned by *Poa* (Figure S2). *Bromus*, *Poa*, and *Solidago* exhibited no directional feedbacks (Figure 1; Tables S7–S9), although *Poa* germinated less frequently in sterile soil relative to its own soil (Figure S3, Table S8). Using an alternative analysis, we detected 21 directional feedbacks among the 42 pairwise comparisons (Figure S3).

### 3.2 Plant‐induced changes in fungal community composition

We characterized fungal communities in soil by sequencing the ITS2 rRNA gene region. We obtained 73,548,493 total fungal reads, with a median sequencing depth per sample of 478,604 reads. After filtering, trimming, and removing chimeras using DADA2, we were left with 62,353,703 (84.8%) reads, which belonged to 3,959 unique taxa. We excluded one sample of Day 0 (preconditioning) soil from further analysis because the sample had fewer than 15,000 reads, indicating a possible sequencing error.

**Figure 2:**
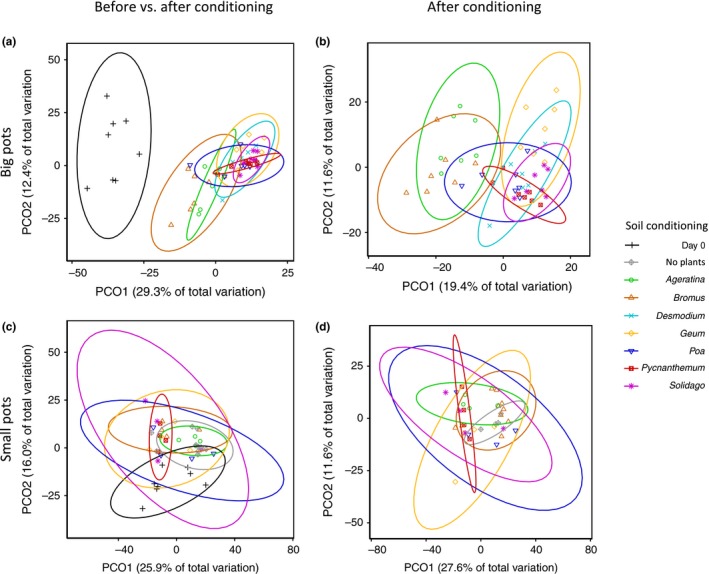
Fungal community differentiation by plant species identity. PCoAs show fungal community differences between plant species in (a) big pots, with comparison to preconditioning (Day 0) soil; (b) big pots, comparing only postconditioning fungal composition; (c) small pots with preconditioning (Day 0) soil; and (d) small pots, comparing only postconditioned soils of each plant species. Each point represents the fungal community in one pot. Colors and shapes correspond to different host plant species or a control in which fungal communities developed without a plant host; ellipses represent 95% confidence levels. Preconditioned soils differed from postconditioned soils in both big and small pots (PERMANOVA; *p* < .05; Tables S10 and S11). In big pots, postconditioning fungal communities were distinct among all species pairs (PERMANOVA; *p* < .05) but this was not the case for fungal communities in small pots (PERMANOVA; *p* > .05; Tables S12 and S13)

Because we detected an interaction between the effect of pot size and plant identity on fungal communities (PERMANOVA; Pseudo‐*F*_5,69_ = 1.72; *p*_adj_ < .001), further analyses on soil fungi were conducted for each pot size separately. In both big and small pots, fungal community composition in soils conditioned by each plant species differed from Day 0 soils (Figure 2a,c; Tables S10 and S11). Excluding Day 0, we found that postconditioning fungal communities differed among soils conditioned by different plant species (Figure 2b,d; Table S12). For large pots, fungal communities were distinct across 18 of the 21 species pairs (Table S13). In small pots, post hoc tests revealed that plant identity did not yield distinct fungal communities after correcting for multiple tests (Table S13). We tested the assumption of homogeneity of variance and found that community dispersion did not differ between any pairwise comparisons of species in big pots (Table S13). Significant differences in dispersion for five pairs in small pots were no longer considered significant following correction for multiple tests (Table S13).

Finally, ASV richness did not differ among soils conditioned by different plant species or in different pot sizes (Figure S4; host species: *F*_6,60_ = 2.14, *p* = .062; pot size: *F*_1,60_ = 0.6957, *p* = .418; host species*pot size: *F*_5,60_ = 0.840, *p* = .523).

### 3.3 Associating fungal communities and feedbacks

**Figure 3:**
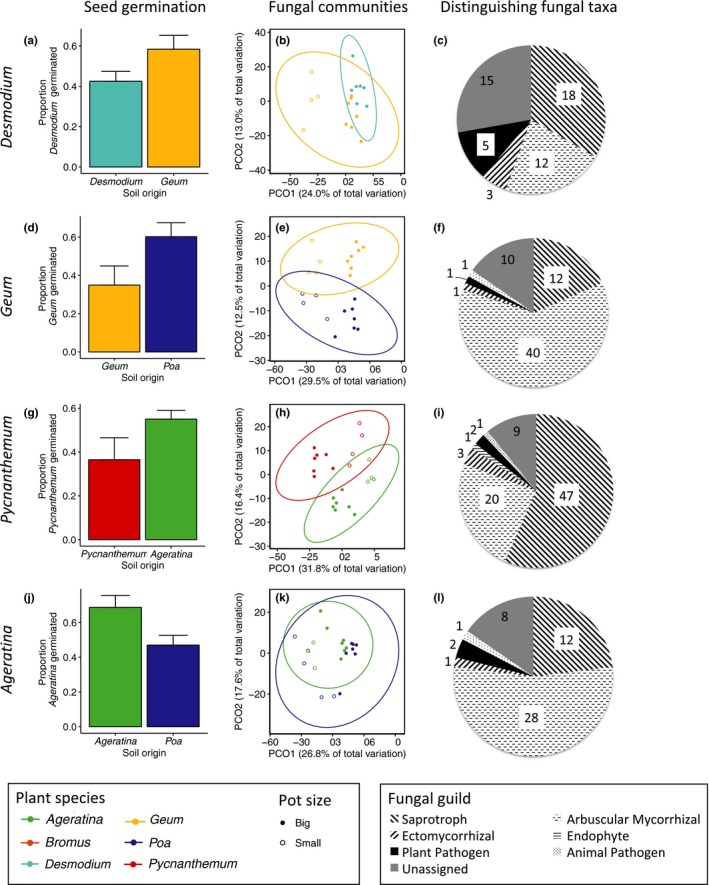
Germination success, fungal community composition, and distinguishing fungal taxa (as identified by FUNGuild) associated with four of the eight statistically significant directional PSFs. Germination trials (left column) included seeds exposed to soils previously conditioned by plants in big and small pots (see Section 2.2); ordinations (middle column) include soils from both big pots (solid symbols) and small pots (hollow symbols). (a) *Desmodium* seeds germinated less in conspecific soil compared to *Geum*‐conditioned soil (negative PSF); (b) *Desmodium* and *Geum* contained distinct fungal soil communities; and (c) 53 fungal ASVs, five of which are potential pathogens, were enriched in *Desmodium* (conspecific) soils relative to *Geum* (heterospecific) soils; (d) *Geum* seeds experienced a negative PSF compared with *Poa*‐conditioned soil; (e) *Geum* and *Poa* soils contained distinct fungal communities; and (f) 65 ASVs, one a potential pathogen, were enriched in *Geum* soil relative to Poa; (g) *Pycnanthemum* seeds experienced greater germination success in *Ageratina* soil; (h) *Pycnanthemum* and *Ageratina* soils contained marginally distinct fungal communities (*p* = .056); and (i) relative to *Ageratina* soil, 83 ASVs were enriched in *Pycnanthemum* soil, including two potential pathogens; (j) *Ageratina* seeds experienced a positive PSF, which was also associated with (k) soil communities that differed in fungal composition between *Ageratina* and *Poa*, and (l) including enrichment of two potential pathogens and 28 potential mutualist ASVs in *Ageratina* soil relative to *Poa*. Ellipses reflect 95% confidence levels. Results from germination tests are contained in Tables S6–S12; PERMANOVA results for fungal communities are found in Table S14. Identities of potential pathogens are reported in Table 1. For the four additional negative PSFs, see Figure S5

Soil fungal community composition differed between pairs of plant species involved in six of the seven negative PSFs and in the positive PSF (Figure 3; Figure S5 and Table S14). When we compared fungal composition differences with PSF values to determine whether community‐wide fungal changes were associated with PSFs, we found that soils with more distinct fungal communities were marginally associated with the magnitude of PSFs (*r*_s_ = .73; *S* = 22, *p* = .046; Figure S6), although the relationship weakens and is no longer marginally significant for negative PSFs only (*r*_s_ = .71, *p* = .088). In both pot sizes, many pairwise differences in fungal community composition emerged among pairs of soils for which we found no directional PSFs on germination (Table S13).

We used a DESeq2 differential abundance analysis (alpha = 0.001) to determine which ASVs differed in the presence and abundance between soils conditioned by different plant species involved in PSFs. Fungi enriched in conspecific‐conditioned soils involved a wide variety of guilds and taxa, only a few of which were identified as pathogens (Figures 3 and S5; Table 1). Thirteen ASVs which distinguished soils involved in feedbacks were classified by FUNGuild as potential plant pathogens (Table 1). While the number of pathogens enriched in conspecific soils ranged from one to five (Figures 3 and S5), seven of the thirteen putative pathogens were enriched in conspecific soils of multiple plant species (Table 1).

**Table 1:** ASVs that differed in abundance between soils involved in at least one of the eight PSFs and that were identified as potential pathogens by FUNGuild

Taxonomy	Potential guild(s)	Species tested for PSF(s)	Enriched in which partner's soil
*Cylindrocladiella* sp.	Plant pathogen	*Desmodium* in *Geum* *Desmodium* in *Solidago* *Geum* in *Poa*	*Desmodium* (conspecific) *Desmodium* (conspecific) *Poa* (heterospecific)
*Didymella* sp. I	Animal pathogen Plant pathogen Unidentified saprotroph	*Desmodium* in *Geum* *Desmodium* in *Pycnanthemum* *Geum* in *Poa* *Pycnanthemum* in *Bromus*	*Desmodium* (conspecific) *Desmodium* (conspecific) *Poa* (heterospecific) *Pycnanthemum* (conspecific)
*Didymella* sp. II	Animal pathogen Plant pathogen Unidentified saprotroph	*Pycnanthemum* in *Ageratina*	*Pycnanthemum* (conspecific)
*Entorrhiza* sp.	Plant pathogen	*Desmodium* in *Geum* *Geum* in *Poa*	*Desmodium* (conspecific) *Poa* (heterospecific)
Helotiaceae sp. I	Ectomycorrhizal Fungal parasite Plant pathogen Wood saprotroph	*Desmodium* in *Bromus* *Desmodium* in *Pycnanthemum* *Geum* in *Poa*	*Bromus* (heterospecific) *Desmodium* (conspecific) *Poa* (heterospecific)
*Helotiaceae* sp. I	Ectomycorrhizal Fungal parasite Plant pathogen Wood saprotroph	*Geum* in *Poa*	*Poa* (heterospecific)
*Helotiaceae* sp. III	Ectomycorrhizal Fungal parasite Plant pathogen Wood saprotroph	*Pycnanthemum* in *Ageratina*	*Pycnanthemum* (conspecific)
*Pleosporaceae*	Endophyte Lichen parasite Plant pathogen Unidentified saprotroph	*Desmodium* in *Bromus* *Desmodium* in *Pycnanthemum* *Desmodium* in *Solidago* *Ageratina* in *Poa*	*Desmodium* (conspecific) *Pycnanthemum* (heterospecific) *Solidago* (heterospecific) *Ageratina* (conspecific)
*Helotiaceae* sp. I	Ectomycorrhizal Fungal parasite Plant pathogen Wood saprotroph	*Desmodium* in *Geum*	*Desmodium* (conspecific)
*Helotiaceae* sp. II	Ectomycorrhizal Fungal parasite Plant pathogen Wood saprotroph	*Desmodium* in *Geum*	*Desmodium* (conspecific)
*Macrophomina* sp.	Plant pathogen	*Geum* in *Poa*	*Poa* (heterospecific)
*Thanatephorus* sp.	Plant pathogen	*Desmodium* in *Bromus* *Pycnanthemum* in *Bromus*	*Bromus* (heterospecific) *Bromus* (heterospecific)
*Veronaea* sp.	Plant pathogen	*Desmodium* in *Bromus* *Desmodium* in *Geum* *Desmodium* in *Pycnanthemum* *Desmodium* in *Solidago* *Geum* in *Poa* *Ageratina* in *Poa*	*Desmodium* (conspecific) *Geum* (heterospecific) *Pycnanthemum* (heterospecific) *Solidago* (heterospecific) *Geum* (conspecific) *Ageratina* (conspecific)

Note

Taxonomy listed is the taxonomy used by FUNGuild to assign guild information; in some cases, multiple ASVs have the same taxonomic assignment. FUNGuild provided multiple potential guilds other than plant pathogen, which are included in the Potential guild(s) column. All FUNGuild guild assignments were assigned a confidence level of Probable. For full citations, see Table S2. The first species listed in the Species tested column is the focal seed species, the second is the identity of the plant that conditioned the soil in which it was buried. The last column indicates whether the pathogen was enriched in conspecific soil or in the soils conditioned by the heterospecific partner.

## 4 DISCUSSION

Our primary goal in this study was to assess whether PSFs operate at the seed stage. We also wanted to link PSF experiments with metagenomics data to characterize the magnitude of microbial community differentiation that accompanies PSFs, and expose the functional groups and identity of fungi that may play a key role in driving directional feedbacks.

Overall, we found that negative PSFs reduced germination in conspecific soils for three focal species, in seven different pairwise combinations (Figure 1). As expected, fungal communities diverged over time and among host plant species (Figures 2 and 3); however, plants that conditioned distinct fungal communities did not always exhibit directional PSFs. Further, our results suggest that AMF and saprophytic fungi comprise the majority of fungi distinguishing conspecific from heterospecific soils; often only one pathogen was identified as enriched in conspecific soils. The identity of distinguishing pathogens depended on the identity of both plant species in the pairing (Table 1), revealing that plant species were not characterized by a particular pathogen. Pathogens, too, were typically enriched in more than one plant species’ soil. If these patterns hold true in natural systems, the suite of pathogens a plant accrues may collectively mediate numerous PSFs on seeds, with feedbacks caused by different pathogens depending on the identity of neighboring species.

### 4.1 PSFs on seed germination

Three different seed species experienced negative PSFs in our study. Of those species, *Desmodium* seeds accounted for half of the pairwise feedbacks we observed, germinating better in soil conditioned by four different partner species than in conspecific‐conditioned soil (Figures 1 and S2). Traits of *Desmodium* seeds may make them more susceptible to pathogen‐mediated mortality than other seed species we tested. For instance, *Desmodium* is the only one of our focal species characterized by physical dormancy (Table S1), a form of dormancy that Dalling, Davis, Schutte, and Arnold (2011) predicted corresponds to fewer chemical defenses devoted to pathogen protection. *Desmodium* seeds are also the heaviest of the species we tested, consistent with studies of (albeit larger) tropical tree seeds that show large size can be linked to pathogen susceptibility (Pringle, Álvarez‐Loayza, & Terborgh, 2007). In contrast, Lebrija‐Trejos, Reich, Hernández, and Wright (2016) found that large‐seeded tree species are less susceptible to conspecific negative density dependence (CNDD); however, their study examined survivorship of seedlings after one year. High risk to pathogens as seeds and low CNDD as seedlings are not mutually exclusive: *Desmodium* seeds may experience high rates of pathogen‐induced mortality, but seeds that survive may be less prone to negative feedbacks as seedlings or mature plants.

While physical traits defend against pathogens, rapid germination allows seeds to escape pathogens (Beckstead, Meyer, Molder, & Smith, 2007; Dalling et al., 2011). In our study, *Bromus* and *Poa* were not vulnerable to pathogen‐mediated PSFs, possibly because they germinated before we exhumed the seed packets. Our data are not sufficient to test trait‐based theories, but given that life history trade‐offs between number, size, and defense traits of seeds influence susceptibility to pathogen attack (Dalling et al., 2011), further research on these traits may allow us to predict which species will be prone to negative PSFs.

We also observed a positive PSF, as *Ageratina* seeds germinated on average 22% more in conspecific soil than in *Poa*‐conditioned soil (Figure 3). Generally, positive PSFs on biomass are attributed to host‐specific mutualists, in particular arbuscular mycorrhizal fungi (AMF; Bever, Westover, & Antonovics, 1997; Callaway, Thelen, Rodriguez, & Holben, 2004). Consistent with this, AMF comprised the majority of ASVs enriched in *Ageratina*‐conditioned soil relative to *Poa*‐conditioned soil (Figure 3). However, this was also the case for *Geum*‐conditioned soil relative to *Poa*, even though *Geum* exhibited a negative PSF (Figure 3). Moreover, the effects of mycorrhizae on germination, while not well understood, are generally negative for the seeds of herbaceous plants (Maighal, Salem, Kohler, & Rillig, 2016; Varga, 2015). *Ageratina* seeds did not differ in germination success between sterile and conspecific soil (Figure S2), suggesting that the positive feedback we observed was due to a negative effect of *Poa*‐conditioned soil on *Ageratina* germination, rather than a positive effect of microbes on *Ageratina* soil. This might occur if pathogens prevalent in *Poa‐*conditioned soil lack host specificity, opportunistically attacking *Ageratina* seeds.

For the majority of species pairs, PSFs on seed germination were neutral, similar to Rutten, Prati, Hemp, and Fischer (2016) and Dudenhöffer et al. (2018). It is tempting to conclude that seeds are less prone to negative PSFs than seedlings, as observed for conspecific negative density dependence more generally (Comita et al., 2014); however, we believe the number and magnitude of directional feedbacks in our study are a conservative estimate for several reasons. First, using both big pot soils (in which fungal communities were distinct among plant species) along with small pot soils (in which we did not detect strong microbial differentiation among plant species) as replicates in our feedback experiment likely reduced the magnitude (and therefore number) of feedbacks we observed. Indeed, *Desmodium*, the species for which we observed the most PSFs, was also the only species with exclusively large pot soils in the feedback portion of the study (Table S1). Second, seed microbiota includes protective microbes and mycoparasites (Nelson, 2017). Because we did not sterilize for an extended period, surviving microbes that protect seeds from pathogen attack may have reduced pathogen‐mediated seed mortality disproportionately in conspecific soils where specialized pathogens accumulate. Finally, it is possible that our conditioning procedure did not favor seed pathogens. In natural systems, seeds banked in the soil support pathogens that target the seed stage (e.g., Beckstead et al., 2007). Although seeds can certainly succumb to host‐specialized pathogens in soils near adult plants (Gallery et al., 2007), it is also plausible that fungal pathogens accumulating around roots of plants during the conditioning phase may not attack seeds. PSFs on seeds may occur more frequently if soil in feedback experiments is conditioned by seeds, not adult plants. Refining our approaches to PSFs in the laboratory, extending seed‐PSF studies to the field, and increasing our understanding of the fungal communities involved (see below) will be necessary to further expose the frequency of PSFs on seeds and their potential importance for plant species coexistence.

### 4.2 Fungal composition in conditioned soils

Consistent with other work showing that plant species develop distinct soil microbial communities (Bever et al., 2012, 1997), we found that fungal composition differed according to plant identity (for plants in big pots) and from the baseline (Time 0) soil fungal community (Figure 2a,c). However, following three‐month conditioning time, no differences in fungal community composition were detected in pairwise tests among species‐conditioned soils in small pots. This is despite the fact that microbial succession occurred, as evidenced by compositional changes between Day 0 soils and final community composition in soils of each species, even in the control pots with no plants (Figure 2c, Table S11). Because differences in fungal compositional between pot sizes were not driven by differences in ASV richness (Figure S4), we speculate that pot size altered abiotic conditions such as temperature or soil moisture, which can shape microbial community composition (Bezemer et al., 2006; Harrison & Bardgett, 2010; Kaisermann, Vries, Griffiths, & Bardgett, 2017). The fact that pot size can slow or inhibit the soil conditioning process provides a cautionary note for comparing results from plant–soil feedback studies using different methods, or conducted under different environmental conditions (Heinze, Sitte, Schindhelm, Wright, & Joshi, 2016). Importantly, identifying whether abiotic conditions alter the degree to which plants influence their soil communities, or the magnitude and direction of PSFs on germination will facilitate more accurate predictions for impacts of climate change on plant community diversity.

Although plants must develop host‐specialized microbial communities for feedbacks to occur (Bever et al., 1997), rarely are fungal seed pathogens strictly species‐specific (Gallery et al., 2007; Hersh et al., 2012; Sarmiento et al., 2017). We, too, saw overlap in the identity of fungal pathogens enriched in soils conditioned by different species (Table 1). Even within one plant species, the identity of pathogens enriched in conspecific soil relative to heterospecific soil depended on the identity of the heterospecific plant partner (e.g., *Pycnathemum* with *Ageratina* vs. *Bromus*, Table 1). This suggests that the taxa that distinguish plant‐conditioned microbial communities—and potentially drive negative PSFs—are a function of pairwise species interactions rather than characteristic of the conditioning host species, per se. If this is true in natural systems, plants that attract diverse fungal pathogens based on root architecture or other traits (Semchenko et al., 2018) may be prone to PSFs with more species partners and potentially play a keystone role in maintaining local plant diversity via PSFs. In the future, linking next‐generation sequencing of fungi in seeds and soils with culturing approaches that allow for direct tests of Koch's postulates will improve our ability to implicate specific pathogens governing negative PSFs on germination, although our data suggest that pathogen identity and function will depend on species pairs (or neighborhoods) and environmental conditions.

Contrary to our prediction, the magnitude of fungal community differentiation did not predict the magnitude of negative PSFs, and in all cases, pathogens comprised a small proportion of the ASVs differentiating conspecific soils from heterospecific soils (Figure 3). On the one hand, our results may demonstrate that pathogens play a minor role in driving negative PSFs. Indeed, negative PSFs ultimately reflect the net effect of multiple positive and negative microbial interactions operating simultaneously, including interactions among other soil microbiota (e.g., bacteria) not quantified in this study (Bever, Mangan, Alexander, 2015). For PSFs on seeds, such interactions also occur within the seed due to vertically transmitted endophytes (Saikkonen, Faeth, Helander, & Sullivan, 1998).

On the other hand, our results may suggest that “a little pathogen goes a long way,” that is, a single distinguishing pathogen may be sufficient to drive negative feedbacks (e.g., Bever et al., 1997; Packer & Clay, 2000). Of course, the presence of host‐specialized pathogens in soils does not necessarily implicate them as drivers of PSFs we observed. Moreover, because fungi can associate with multiple plant species but have different effects on each (“effective specialization”; Benítez et al., 2013), pathogens present in both conspecific and heterospecific soils (and thus not indicated as “enriched,” or host‐specialized, in our analysis) may play a significant role in generating feedbacks. Though our data cannot discriminate between these explanations, a recent meta‐analysis showed that pathogens are the strongest drivers of negative PSFs, not indirect effects of other fungi (Crawford et al., 2019). Consequently, the fact that we observed PSFs on seeds and identified candidate pathogens from soils in which they were buried offers a productive starting point for pathogenicity tests that in turn provide direct evidence for the governing role, and identity, of pathogens driving negative PSFs on seed germination.

## 5 CONCLUSIONS

For some grassland species, plant–soil feedbacks influence seed germination—a key fitness component for plants—and those PSFs were associated with community‐wide shifts in soil fungi. Whether distinct microbial communities contain keystone, host‐specific pathogens, remains a focus for future research. Notably, we detected no reciprocal feedbacks, that is, in no case did two species germinate more in each others’ soils relative to their own, as might be expected if PSFs on seed germination were a key mechanism promoting coexistence (Crawford et al., 2019). Future PSF studies that test multiple species across defense syndromes (Dalling et al., 2011) under different environmental conditions will increase our ability to predict when and if seeds will succumb to PSFs, and if they play a role in maintaining aboveground plant diversity. Ultimately, characterizing negative PSFs on seeds, particularly in the field, and predicting their magnitude based on seed traits could prove useful for maximizing diversity in restoration (Kardol & Wardle, 2010; Kremer, Caesar, & Souissi, 2006) and predicting the impact of global changes on PSFs (van der Putten et al., 2013).

## CONFLICT OF INTEREST

None declared.

## AUTHORS' CONTRIBUTIONS

CDC and ECM designed the study; ECM collected the data; CDC, ECM, and GGP analyzed data; CDC, ECM, and GGP wrote the manuscript.

## Data Availability

Data available from the Sequence Read Archive at NCBI (accession number: PRJNA553564) and Dryad Digital Repository (https://doi.org/10.5061/dryad.7nm4970).
