# Supplementary material for: Plant‐driven changes in soil microbial communities influence seed germination through negative feedbacks
Source: Ecol Evol. 2019 Jul 25;9(16):9298–311. doi: 10.1002/ece3.5476 (PMC6706191; doi:10.1002/ece3.5476)
Supplement: Supplementary file 2 [file ECE3-9-9298-s002.docx]

SUPPLEMENTARY INFORMATION

*Miller, E.C., Perron, G.G., Collins, C.D. Plant-driven changes in soil microbial communities influence seed germination through negative feedbacks*

Tables

**Table S1. Life history and seed traits of** focal plant species

**Table S2.** Fungal ASVs enriched in soil of species relative to a partner species in PSF based on DESeq2 differential abundance analysis. **Excel table in separate file.**

**Table S3.** Linear model for *Desmodium* germination in conspecific vs heterospecific soil

**Table S4.** Linear model for *Pycnanthemum* germination in conspecific vs heterospecific soil

**Table S5.** Linear model for *Geum* germination in conspecific vs heterospecific soil

**Table S6.** Linear model for *Ageratina* germination in conspecific vs heterospecific soil

**Table S7.** Generalized linear model for *Bromus* germination in conspecific vs heterospecific soil

**Table S8.** Linear model for *Poa* germination in conspecific vs heterospecific soil

**Table S9.** Linear model for *Solidago* germination in conspecific vs heterospecific soil

**Table S10.** Fungal composition in soils conditioned among seven species and Day 0 soils

**Table S11.** Post-hoc tests of soil fungi composition between soils conditioned by each plant species and Day 0 soils

**Table S12**. Fungal composition after three months conditioning by seven plant species

**Table S13.** Post-hoc, pairwise tests of post-conditioning soil fungi communities large and small pots separately.

**Table S14.** Comparison of fungal communities only for species’ pairs with directional PSFs

Figures

**Figure S1**. Proportion of seeds germinating prior to exhuming seed bags

**Figure S2**. Seed germination in conspecific, heterospecific and sterile soils

**Figure S3.** Statistically significant PSFs as indicated by one-sample t-tests

**Figure S4.** Fungal richness according to plant identity and pot size

**Figure S5.** Germination success, fungal community composition, and distinguishing fungi for four of eight species pairs exhibiting PSFs.

**Figure S6.** Relationship between fungal community differences and feedback magnitudes for the eight pairs of soils that experienced significant feedbacks

**Table S1.** Sample sizes and traits of focal plants used in the study. NA indicates ecotype was not available when ordering seeds. Frequency in landscape was determined as the proportion of 1 m^2^ plots (n = 585) containing each species (Collins, *unpublished data*). Seed mass is the average of 25 randomly selected seeds. PD^[[1]](#footnote-1)^: physiological dormancy, PY: physical dormancy, ND: non-dormant. Last two columns show pot size numbers for each species, for each phase of the study.

| **Attributes of Focal Plants** | | | | | | | | | |
| --- | --- | --- | --- | --- | --- | --- | --- | --- | --- |
| *Species* | *Growth form* | *Seed ecotype* | *Frequency in landscape* | *Average seed mass (mg)* | *Dispersal mode* | | *Dormancy* | *Big pot replicates:*  *conditioning/*  *metagenomics/*  *feedback* | *Small pot replicates:*  *conditioned/*  *metagenomics/*  *feedback* |
| *Bromus inermis* | Grass | NA | 0.403 | 3.04 | Wind^[[2]](#footnote-2)^ | | ND^9^ | 21/6/3 | 14/5/2 |
| *Desmodium illinoense* | Forb (legume) | Whiteside Co., IL | 0.232 | 6.67 | Zoochory^[[3]](#footnote-3)^  (Seed Pods) |  | PY ^10^ | 35/8/5 | 0/0/0 |
| *Ageratina altissima* | Forb | NA | 0.171 | 0.138 | Wind^[[4]](#footnote-4)^ | | PD^10^ | 21/6/3 | 14/5/2 |
| *Geum canadense* | Forb | White Avens, PA | 0.455 | 1.490 | Wind^[[5]](#footnote-5)^ | | ND^1^ | 21/6/3 | 14/5/2 |
| *Poa pratensis* | Grass | NA | 0.405 | 0.326 | Zoochory^[[6]](#footnote-6)^ | | ND^10^ | 21/6/3 | 14/5/2 |
| *Pycnanthemum tenuifolium* | Forb | Whiteside Co., IL | 0.024 | 0.035 | Wind^[[7]](#footnote-7)^ | | PD^11^ | 21/6/3 | 14/5/2 |
| *Solidago canadensis* | Forb | NA | 0.060 | 0.070 | Wind^[[8]](#footnote-8)^ | | PD^12^ | 21/6/3 | 14/5/2 |

**Table S2.** Excel table of ASVs and functional guild assignments (separate .xls file)

**Table S3.** Results for General Linear Model for *Desmodium* seed germination in soils conditioned by heterospecifics relative to soils conditioned by *Desmodium* (overall model: *F*_(7,31)_ =2.29, *P*=0.05)*.* Factors were re-leveled so that *Desmodium* (conspecific) soil was the baseline; consequently, germination in each heterospecific soil was compared to germination in *Desmodium*.

|  | **Proportion *Desmodium* germinated** | | |
| --- | --- | --- | --- |
| *Predictors* | *Estimates* | *CI* | *p* |
| (Intercept) | 0.43 | 0.32 – 0.53 | **<0.001** |
| *Ageratina* | 0.03 | -0.11 – 0.17 | 0.671 |
| *Bromus* | 0.17 | 0.03 – 0.31 | **0.028** |
| *Geum* | 0.16 | 0.02 – 0.30 | **0.035** |
| *Poa* | 0.09 | -0.05 – 0.23 | 0.238 |
| *Pycnanthemum* | 0.15 | 0.01 – 0.29 | **0.050** |
| *Solidago* | 0.16 | 0.02 – 0.30 | **0.035** |
| Sterile | 0.24 | 0.09 – 0.40 | **0.003** |
| Observations | 39 | | |
| R^2^ / adjusted R^2^ | 0.341 / 0.192 | | |

**Table S4.** Results for General Linear Model for *Pycnanthemum* seed germination in soils conditioned by heterospecifics relative to soils conditioned by *Pycnanthemum* (overall model: *F*_(7,32)_ =3.41, *P*=0.017)*.* Factors were re-leveled so that *Pycnanthemum* (conspecific) soil was the baseline; consequently, germination in each heterospecific soil was compared to germination in *Pycnanthemum.*

|  | **Proportion *Pycnanthemum* germinated** | | |
| --- | --- | --- | --- |
| *Predictors* | *Estimates* | *CI* | *p* |
| (Intercept) | 0.37 | 0.26 – 0.47 | **<0.001** |
| *Ageratina* | 0.19 | 0.03 – 0.34 | **0.025** |
| *Bromus* | 0.19 | 0.03 – 0.34 | **0.024** |
| *Desmodium* | 0.08 | -0.07 – 0.24 | 0.302 |
| *Geum* | 0.02 | -0.13 – 0.17 | 0.798 |
| *Poa* | 0.00 | -0.15 – 0.16 | 0.970 |
| *Solidago* | 0.00 | -0.15 – 0.16 | 0.967 |
| Sterile | -0.12 | -0.27 – 0.04 | 0.140 |
| Observations | 40 | | |
| R^2^ / adjusted R^2^ | 0.427 / 0.302 | | |

**Table S5.** Results for General Linear Model for *Geum* seed germination in soils conditioned by heterospecifics relative to soils conditioned by *Geum* (overall model: *F*_(7,31)_ =2.96, *P*=0.02)*.* Factors were re-leveled so that *Geum* (conspecific) soil was the baseline; consequently, germination in each heterospecific soil was compared to germination in *Geum.*

|  | **Proportion *Geum* germinated** | | |
| --- | --- | --- | --- |
| *Predictors* | *Estimates* | *CI* | *p* |
| (Intercept) | 0.35 | 0.19 – 0.51 | **<0.001** |
| *Ageratina* | -0.03 | -0.26 – 0.19 | 0.771 |
| *Bromus* | 0.10 | -0.13 – 0.32 | 0.401 |
| *Desmodium* | 0.17 | -0.06 – 0.39 | 0.154 |
| *Poa* | 0.25 | 0.03 – 0.48 | **0.035** |
| *Pycnanthemum* | 0.16 | -0.06 – 0.39 | 0.165 |
| *Solidago* | 0.21 | -0.02 – 0.43 | 0.078 |
| Sterile | -0.18 | -0.42 – 0.05 | 0.139 |
| Observations | 39 | | |
| R^2^ / adjusted R^2^ | 0.400 / 0.265 | | |

**Table S6.** Results for General Linear Model for *Ageratina* seed germination in soils conditioned by heterospecifics relative to soils conditioned by *Ageratina* (overall model: *F*_(7,31)_ =1.89, *P*=0.10)*.* Factors were re-leveled so that *Ageratina* (conspecific) soil was the baseline; consequently, germination in each heterospecific soil was compared to germination in *Ageratina*.

|  | **Proportion *Ageratina* Germinated** | | |
| --- | --- | --- | --- |
| *Predictors* | *Estimates* | *CI* | *p* |
| (Intercept) | 0.69 | 0.57 – 0.80 | **<0.001** |
| *Bromus* | -0.01 | -0.17 – 0.15 | 0.923 |
| *Desmodium* | -0.11 | -0.27 – 0.05 | 0.195 |
| *Geum* | -0.05 | -0.21 – 0.11 | 0.523 |
| *Poa* | -0.22 | -0.38 – -0.06 | **0.013** |
| *Pycnanthemum* | -0.11 | -0.27 – 0.05 | 0.186 |
| *Solidago* | 0.02 | -0.14 – 0.18 | 0.834 |
| Sterile | -0.00 | -0.17 – 0.17 | 1.000 |
| Observations | 39 | | |
| R^2^ / adjusted R^2^ | 0.300 / 0.141 | | |

**Table S7.** Results for Generalized Linear Model with binomial error of *Bromus* seed germination in soils conditioned by heterospecific plants relative to soils conditioned by *Bromus*. Factors were re-leveled so that *Bromus* (conspecific) soil was the baseline; consequently, germination in each heterospecific soil was compared to germination in *Bromus*.

|  | **Proportion *Bromus* seeds germinated** | | |
| --- | --- | --- | --- |
| *Predictors* | *Odds Ratios* | *CI* | *p* |
| (Intercept) | 3.46 | 0.42 – 28.36 | 0.247 |
| *Ageratina* | 1.35 | 0.06 – 30.52 | 0.850 |
| *Desmodium* | 0.61 | 0.04 – 10.29 | 0.734 |
| *Geum* | 1.35 | 0.06 – 30.52 | 0.850 |
| *Poa* | 1.08 | 0.05 – 21.72 | 0.962 |
| *Pycnanthemum* | 0.74 | 0.04 – 13.08 | 0.839 |
| *Solidago* | 1.15 | 0.06 – 24.06 | 0.926 |
| Sterile | 1.64 | 0.05 – 51.90 | 0.780 |
| Observations | 39 | | |
| Cox & Snell's R^2^ / Nagelkerke's R^2^ | 0.016 / 0.248 | | |

**Table S8.** Results for General Linear Model for *Poa* seed germination in soils conditioned by heterospecifics relative to soils conditioned by *Poa* (overall model: *F*_(7,31)_ =3.69, *P*=0.005)*.* Factors were re-leveled so that *Poa* (conspecific) soil was the baseline; consequently, germination in each heterospecific soil was compared to germination in *Poa.*

|  | **Proportion *Poa* germinated** | | |
| --- | --- | --- | --- |
| *Predictors* | *Estimates* | *CI* | *p* |
| (Intercept) | 0.51 | 0.34 – 0.69 | **<0.001** |
| *Ageratina* | -0.14 | -0.39 – 0.11 | 0.281 |
| *Bromus* | -0.18 | -0.42 – 0.07 | 0.174 |
| *Desmodium* | -0.14 | -0.39 – 0.11 | 0.274 |
| *Geum* | 0.09 | -0.16 – 0.33 | 0.497 |
| *Pycnanthemum* | 0.17 | -0.07 – 0.42 | 0.176 |
| *Solidago* | 0.05 | -0.19 – 0.30 | 0.667 |
| Sterile | -0.39 | -0.65 – -0.13 | **0.007** |
| Observations | 39 | | |
| R^2^ / adjusted R^2^ | 0.455 / 0.331 | | |

**Table S9.** Results for General Linear Model for *Solidago* seed germination in soils conditioned by heterospecifics relative to soils conditioned by *Solidago* (overall model: *F*_(7,31)_ =0.83, *P*=0.57)*.* Factors were re-leveled so that *Solidago* (conspecific) soil was the baseline; consequently, germination in each heterospecific soil was compared to germination in *Solidago.*

|  | **Proportion *Solidago* germinated** | | |
| --- | --- | --- | --- |
| *Predictors* | *Estimates* | *CI* | *p* |
| (Intercept) | 0.56 | 0.45 – 0.67 | **<0.001** |
| *Ageratina* | -0.04 | -0.19 – 0.12 | 0.661 |
| *Bromus* | -0.07 | -0.23 – 0.08 | 0.365 |
| *Desmodium* | -0.00 | -0.16 – 0.15 | 0.977 |
| *Geum* | -0.03 | -0.19 – 0.13 | 0.709 |
| *Poa* | 0.06 | -0.10 – 0.21 | 0.476 |
| *Pycnanthemum* | -0.07 | -0.23 – 0.09 | 0.378 |
| Sterile | -0.11 | -0.28 – 0.05 | 0.192 |
| Observations | 39 | | |
| R^2^ / adjusted R^2^ | 0.157 / -0.033 | | |

**Table S10.** Differences in fungal composition in soils conditioned different plant species; and baseline soils. Results from PERMANOVA with 9999 permutations performed on fungal community data using Morisita-Horn distances calculated from log (x +1) transformed abundance (number of reads) data. The main factor conditioning treatment includes the seven plant species and the Day 0 (unconditioned) soils as levels. Posthoc comparisons between each soils conditioned by each species vs Day 0 (unconditioned) soils are presented in Table S3.

*Factor*  *df MS Pseudo-F P*

**Large Pot Soil**

Conditioning Treatment 7 3522.1 6.3714 **0.0001**

Residual 49 552.8

Total 56

**Small Pot Soil**

Conditioning Treatment 7 1523.4 2.35 **0.0001**

Residual 34 646.9

Total 41

**Table S11.** Pairwise comparisons of fungal community composition between soil conditioned by each plant species and Day 0 soils. Bold-faced *P*-values are those that remained significant following a Benjamin-Hochberg correction for 5% false discovery rate. PERMDISP results show dispersion (multivariate variance) differs between groups. Note that only *Desmodium* was conditioned only in large pots, and “control” soil with no plants were in small pots only.

PERMANOVA PERMDISP

*Pairwise comparison* *t P t P*

**Large Pot Soil**

*Ageratina*, Day 0 2.93 **0.001** 0.47 0.650

*Bromus,* Day 0 2.96 **0.001** 0.77 0.490

*Desmodium*, Day 0 3.79 **0.001** 0.53 0.618

*Geum*, Day 0 2.96 **0.001** 1.00 0.374

*Poa*, Day 0 2.60 **0.001** 0.63 0.585

*Pycnanthemum*, Day 0 3.14 **0.001** 0.93 0.414

*Solidago*, Day 0 3.02 **0.001** 1.02 0.394

**Small Pot Soil**

*Ageratina*, Day 0 2.11 **0.009** 1.15 0.412

*Bromus,* Day 0 2.11 **0.005** 0.23 0.869

*No plant*  Day 0 2.38 **0.001** 0.20 0.884

*Geum*, Day 0 2.06 **0.005** 0.39 0.755

*Poa*, Day 0 1.52 **0.071** 1.34 0.292

*Pycnanthemum*, Day 0 1.87 **0.007** 0.56 0.700

*Solidago*, Day 0 1.74 **0.028** 2.33 0.081

**Table S12.** Fungal composition in soils following three months conditioning (i.e., excluding Day 0 soils). Results are from PERMANOVA with 9999 permutations performed on fungal community data using Morisita-Horn distances calculated from log (x +1) transformed abundance data. The main factor conditioning treatment refers to either plant identity of conditioned soils, or in the case of Small Pot Soil, it also includes control soil in pots with no plants. Because *Desmodium* was conditioned only in large pots, and no-plant soils were only in small plots, the df for Conditioning Treatment are the same for both pot sizes. Posthoc comparisons between each species are presented in Table S13.

*Factor*  *df MS Pseudo-F P*

**Large Pot Soil**

Conditioning Treatment 6 2003.3 3.62 **0.0001**

Residual 42 553.26

Total 48

**Small Pot Soil**

Conditioning Treatment 6 1153 1.72 **0.002**

Residual 27 672.01

Total 33

**Table S13.** Pairwise comparisons of fungal community composition between soil conditioned by each plant species. PERMDISP results show dispersion (multivariate variance) between groups. Bold-faced p-values are those that remained significant following a Benjamin-Hochberg correction for 5% false discovery rate. Note that only *Desmodium* was conditioned only in large pots, and only small pots contained “control” soil with no plants.

PERMANOVA PERMDISP

*Pairwise comparison*  *t P t P*

**Large Pot Soil**

*Bromus*, *Desmodium* 2.39 **<0.001** 1.28 0.29

*Bromus*, *Geum*  2.11 **<0.001** 2.32 0.043

*Desmodium*, *Ageratina* 2.22 **<0.001** 1.89 0.105

*Bromus*, *Pycnanthemum* 1.95 **0.001** 0.61 0.59

*Bromus*, *Solidago* 1.87 **0.001** 1.29 0.296

*Ageratina*, *Pycnanthemum* 1.89 **0.002**  0.34 0.772

*Desmodium*, *Geum* 1.80  **0.005**  3.03 0.012

*Ageratina*, *Geum* 1.76 **0.006** 2.19 0.048

*Geum*, *Pycnanthemum* 1.78 **0.006**  1.71 0.157

*Ageratina*, *Solidago* 1.68 **0.009**  1.12 0.351

*Geum*, *Poa* 1.70 **0.013**  0.611 0.609

*Desmodium*, *Pycnanthemum* 1.45 **0.017**  1.74 0.182

*Desmodium*, *Poa* 1.53 **0.028**  2.06 0.157

*Geum*, *Solidago* 1.45  **0.034** 0.60 0.621

*Desmodium*, *Solidago* 1.32 **0.042**  1.90 0.160

*Bromus*, *Poa* 1.42 **0.045**  1.40 0.265

*Ageratina*, *Poa* 1.44 **0.052**  1.22 0.323

*Bromus*, *Ageratina* 1.35 **0.054**  0.36 0.728

*Poa*, *Solidago* 1.12 0.23 0.02 0.987

*Pycnanthemum*, *Solidago* 1.03 0.31 0.82 0.536

*Poa*, P*ycnanthemum* 0.96 0.45 0.89 0.501

PERMANOVA PERMDISP

*Pairwise comparison*  *t P t P*

**Small Pot Soil**

*Geum*, Control 1.93 0.011 0.44 0.723

*Pycnanthemum*, Control 1.73 0.017 0.54 0.709

*Bromus*, Control 1.60 0.036 0.002 0.999

*Ageratina*, *Pycnanthemum* 1.57 0.052 2.65 0.117

*Bromus*, *Ageratina* 1.57 0.068 1.26 0.320 *Solidago*, Control 1.45 0.078 1.81 0.214

*Ageratina*, *Geum* 1.45 0.108 1.39 0.251

*Bromus*, *Pycnanthemum* 1.36 0.13 1.38 0.248

*Geum*, *Pycnanthemum* 1.34 0.13 0.084 0.945

*Bromus*, *Geum* 1.28 0.17 0.61 0.688

*Ageratina*, *Solidago* 1.23 0.21 4.18 0.030

*Poa*, *Pycnanthemum* 1.16 0.26 1.22 0.144

*Bromus*, *Poa* 1.16 0.27 2.03 0.060

*Geum*, *Poa* 1.15 0.27 0.78 0.489

*Ageratina*, Control 1.12 0.27 0.64 0.692

*Poa*, Control 1.03 0.37 1.11 0.435

*Geum*, *Solidago* 1.02 0.41 1.63 0.314

*Bromus*, *Solidago* 0.99 0.45 3.24 0.031

*Ageratina*, *Poa* 0.96 0.45 2.98 0.060

*Poa*, *Solidago* 0.81 0.64 1.13 0.287

*Pycnanthemum*, *Solidago* 0.77 0.73 2.60 0.029

**Table S14.** Results from PERMANOVA and PERDISP with 9999 permutations performed on a Morisita-Horn distance matrix calculated for abundance data (number of reads) transformed using log (x +1) for each pair of soils for which we detected statistically significant feedbacks. Because replicates for the exposure phase of the feedback trials included soils conditioned by plants contained in both big and small pots, comparisons reported below include soils from both pot sizes. *Desmodium* soils were conditioned in large pots only; in those comparison (e.g., *Desmodium*, *Geum* below), the soils from *Desmodium* were from big pots only but *Geum* includes soils conditioned in both big and small pots. Pot size is designated in ordinations, and figure locations are included in the table below to easily link ordination results with appropriate statistic.

PERMANOVA PERMDISP Figure

*Pairwise comparison*  Psuedo-*F P F P* Location

*Desmodium, Geum*  3.23 0.005 9.18 0.005 Fig. 3b

*Geum, Poa* 2.90 0.012 0.37 0.618 Fig. 3e

*Pycnanthemum, Ageratina* 3.57 0.001 0.11 0.771 Fig. 3h

*Ageratina, Poa*  2.08 0.056 1.49 0.324 Fig. 3k

*Desmodium, Bromus* 5.72 0.001 1.64 0.293 Fig. S5b

*Desmodium, Pycnanthemum* 2.11 0.017 3.02 0.194 Fig. S5e

*Desmodium, Solidago* 1.73 0.043 3.60 0.158 Fig. S5h

*Pycnanthemum, Bromus* 3.79 <0.001 0.38 0.375 Fig. S5k


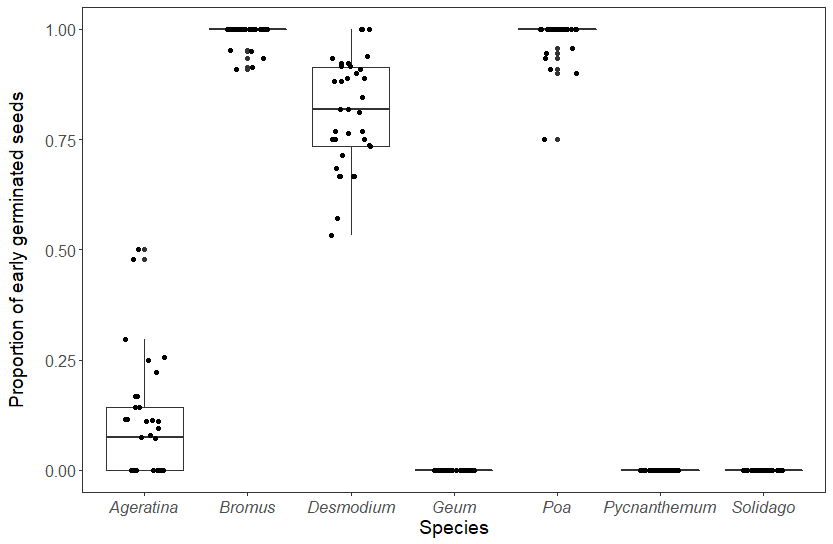


**Figure S1.**  Proportion of seeds for each of seven species that germinated in bags while buried during the feedback experiment. Each bag contained 25 seeds and was buried for three months to expose seeds to soil microbes conditioned by plants. Proportions were calculated using the number of seeds that germinated prior to exhuming the bag, divided by the total number of seeds that germinated during the entire study (tallied after the germination trials). Raw data points are jittered .

**Figure S2:** Germination proportions of seeds after burial in soils under different plant species or sterile soil. Titles reflect identity of the focal seed species that was buried, and black bars show conspecific seed/soil pairings. Error bars reflect standard error. Brackets show significantly different germination success between soil conditioned by a heterospecific partner relative to conspecific soil (*p<0.05, **p<0.01; see Tables S6-S12).


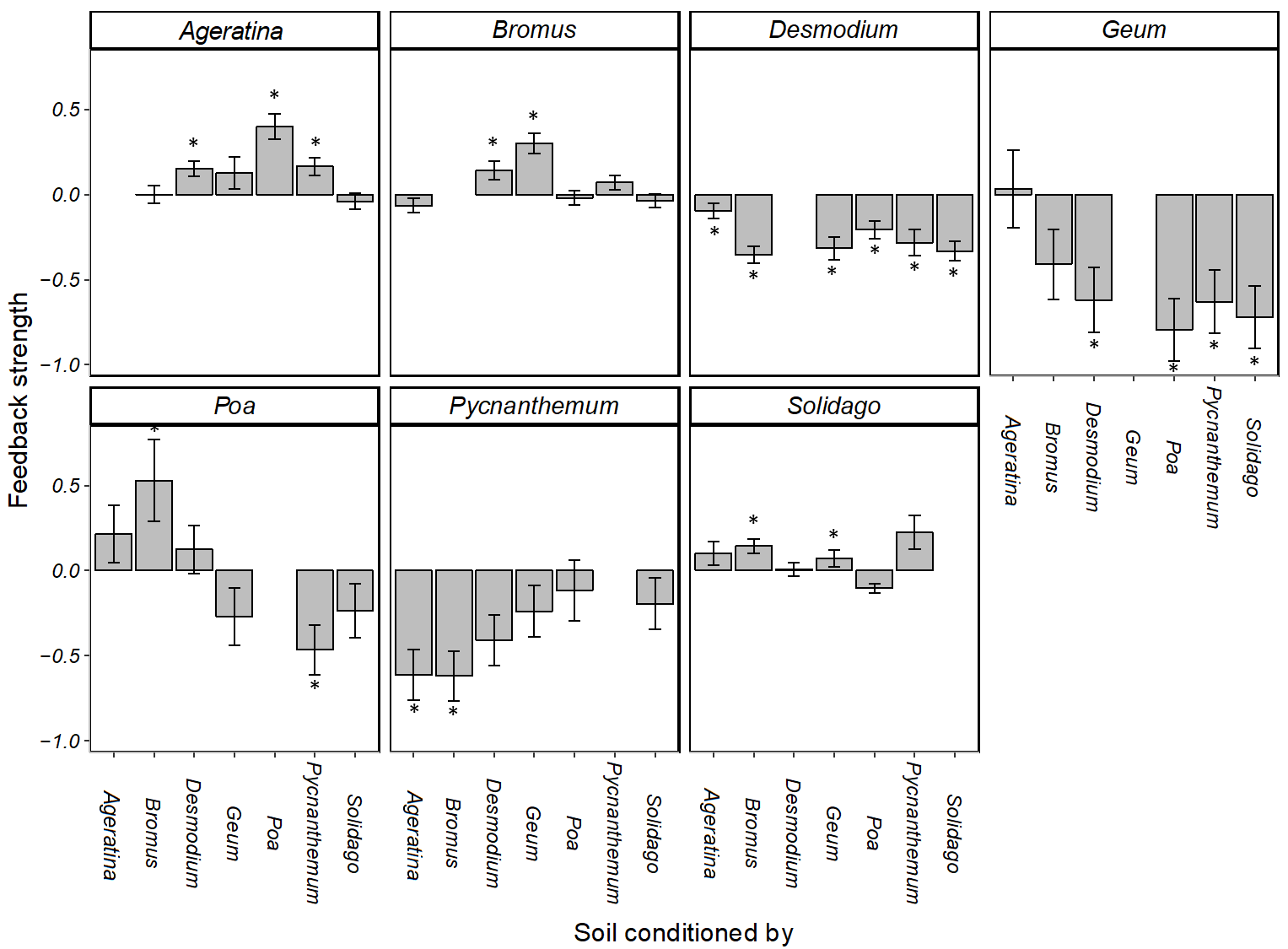


**Figure S3.** Strength of PSF on seeds germinated in conspecific vs. heterospecific soil. Each panel contains results for seeds of a different plant species; x-axis labels reflect the identity of the heterospecific host-plant-conditioned soil. PSFs were calculated as ln(germination in conspecific soil/germination in heterospecific soil). Positive feedback values indicate positive PSFs (higher germination in conspecific soil compared to other species’ or sterile soil) and negative feedback values indicate negative PSFs (lower germination in conspecific soil compared to other species’ or sterile soil). Error bars show standard error and * indicates statistically significant PSFs (*P* < 0.05) based on one-sample t-tests (see Methods) using feedback strength as the response variable.

**Figure S4:** Fungal ASV richness, as measured by number of reads, in soils conditioned by different plant species in big and small pots. Error bars show standard error.

**Figure S5.** Germination success, fungal community composition, and distinguishing fungal taxa (as identified by FUNGuild) associated with conspecific soil from four of the seven negative PSFs. **a)** Reduced germination of *Desmodium* seeds in conspecific soil compared to *Bromus*-conditioned soil was associated with **b)** distinct fungal soil communities conditioned by each plant and **c**) 111 fungal ASVs in conspecific soil, two of which are potential pathogens were enriched in Desmodium soil relative to Bromus. **d)** *Desmodium* seeds experienced reduced germination in conspecific soil compared with *Pycnanthemum*-conditioned soi; **e)** fungal communities differed between these two soils, and **f)** 55 ASVs, one a potential pathogen, were enriched in *Desmodium* soil relative to *Pycnanthemum*-condition soil;. **g)** *Desmodium* seeds also experienced greater germination success in *Solidago* soil, a negative feedback that was associated with **h)** distinct fungal communities and **i)** 83 ASVs enriched in *Desmodium* soil, including two potential pathogens. **j)** *Pycnanthemum* seeds experienced a negative PSF, which was associated with **k)** soil communities that differed in composition between conspecific and *Bromus* soil and **l)** 92 ASVs enriched in conspecific soil, including one potential pathogen. Elipses reflect 95% confidence levels. For the additional four negative PSFs, see Fig. S4. Results from germination tests contained in Tables S6-S12; PERMANOVA results for fungal communities found in Table S13. Identities of potential pathogens are reported in Table 1.

**Figure S6.** Relationship between fungal community differences and feedback magnitudes for the eight pairs of soils that experienced significant feedbacks. The centroid distances between each of two soil fungal communities, while the magnitude of feedback was calculated as absolute value of ln(germination success in conspecific soil/germination success in heterospecific soil). Including the positive PSF between *Ageratina-Poa*, this relationship is marginally significant (r_s_= 0.73; S=22, *P*=0.046); for negative PSFs only (not pictured here), the relationship is not statistically significant (r_s_=0.71, *P*=0.088).

1. Baskin C.C., Baskin J.M. 2014. Seeds: ecology, biogeography, and evolution of dormancy and germination. Second Edition. San Diego: Academic/Elsevier. [↑](#footnote-ref-1)
2. Otfinowski R., Kenkel N.C., Catling P.M. 2007. The biology of Canadian weeds. 134. *Bromus inermis* Leyss. *Canadian Journal of Plant Science* 87(1), 183-198. [↑](#footnote-ref-2)
3. Hilty, J. 2002-2018. Illinois Tick Trefoil. http://www.illinoiswildflowers.info/index.htm [↑](#footnote-ref-3)
4. Cain M.L., Damman H., Muir A. 1998. Seed dispersal and the Holocene migration of woodland herbs. *Ecological Monographs*. 68(3), 325-347. [↑](#footnote-ref-4)
5. Sorenson A.E. 1986. Seed dispersal by adhesion. *Annual Review of Ecological and Systematics*. 17, 443-463. [↑](#footnote-ref-5)
6. Fischer S.F., Poschlod P., Beinlich B. 1996. Experimental studies on the dispersal of plants and animals on sheep in calcareous grasslands. *Jounral of Applied* *Ecology* 33(5), 1206-1222. [↑](#footnote-ref-6)
7. Sheahan, C.M.2012. Plant guide for narrowleaf mountainmint (*Pycnanthemum tenuifolium*). USDA-NRCS, Cape May Plant Materials Center. Cape May, NJ. [↑](#footnote-ref-7)
8. Pavek, P.L.S. 2011. Plant guide for Canada goldenrod (*Solidago canadensis*). USDA-NRC. Pullman, WA.

   ^9^ Yang H., Huang Z., Baskin C.C., Baskin J.M., Cao Z., Zhu X., Dong M. 2009. Responses of caryopsis germination, early seedling growth and ramet clonal growth of *Bromus inermis* to soil salinity. *Plant Soil* 316, 265-275.

   ^10^ Willis C.G., Baskin C.C., Baskin J.M., Auld J.R., Lawrence Venable D., Cavender-Bares J., Donohue K., Rubio de Casas R., The NESCent Germination Working Group. 2014. The evolution of seed dormancy: environmental cues, evolutionary hubs, and diversification of the seed plants. *New Phytologist*. 203, 300-309.

   ^11^ Sullivan G.A., Daley R.H. 1981. Directory to resources on wildflower propogation. National Council of State Garden Clubs, Inc. prepared at Missouri Botanical Garden, St. Louis.

   ^12^ Martinkova, Z., Honek, A., Stolcova J. 1997. The incidence of primary seed dormancy in weed species of the Czech Republic. *Ochrana Rostlin*. 33, 265-279. [↑](#footnote-ref-8)
